# An improved method for the molecular identification of single dinoflagellate cysts

**DOI:** 10.7717/peerj.3224

**Published:** 2017-04-26

**Authors:** Yangchun Gao, Hongda Fang, Yanhong Dong, Haitao Li, Chuanliang Pu, Aibin Zhan

**Affiliations:** 1 Research Center for Eco-Environmental Sciences, Chinese Academy of Sciences, Beijing, China; 2 University of Chinese Academy of Sciences, Chinese Academy of Sciences, Beijing, China; 3 South China Sea Environmental Monitoring Center, State Oceanic Administration, Guangzhou, China

**Keywords:** Dinoflagellate cysts, Harmful algae, Ultrasonic cleaning, PCR inhibitor, Micropipette cleaning

## Abstract

**Background:**

Dinoflagellate cysts (i.e., dinocysts) are biologically and ecologically important as they can help dinoflagellate species survive harsh environments, facilitate their dispersal and serve as seeds for harmful algal blooms. In addition, dinocysts derived from some species can produce more toxins than vegetative forms, largely affecting species through their food webs and even human health. Consequently, accurate identification of dinocysts represents the first crucial step in many ecological studies. As dinocysts have limited or even no available taxonomic keys, molecular methods have become the first priority for dinocyst identification. However, molecular identification of dinocysts, particularly when using single cells, poses technical challenges. The most serious is the low success rate of PCR, especially for heterotrophic species.

**Methods:**

In this study, we aim to improve the success rate of single dinocyst identification for the chosen dinocyst species (*Gonyaulax spinifera*, *Polykrikos kofoidii*, *Lingulodinium polyedrum*, *Pyrophacus steinii, Protoperidinium leonis* and *Protoperidinium oblongum*) distributed in the South China Sea. We worked on two major technical issues: cleaning possible PCR inhibitors attached on the cyst surface and designing new dinoflagellate-specific PCR primers to improve the success of PCR amplification.

**Results:**

For the cleaning of single dinocysts separated from marine sediments, we used ultrasonic wave-based cleaning and optimized cleaning parameters. Our results showed that the optimized ultrasonic wave-based cleaning method largely improved the identification success rate and accuracy of both molecular and morphological identifications. For the molecular identification with the newly designed dinoflagellate-specific primers (18S634F-18S634R), the success ratio was as high as 86.7% for single dinocysts across multiple taxa when using the optimized ultrasonic wave-based cleaning method, and much higher than that (16.7%) based on traditional micropipette-based cleaning.

**Discussion:**

The technically simple but robust method improved on in this study is expected to serve as a powerful tool in deep understanding of population dynamics of dinocysts and the causes and consequences of potential negative effects caused by dinocysts.

## Introduction

More than 200 known marine dinoflagellate species can produce cysts (Matsuoka & Fukuyo, 2000; Wang, 2007). Dinoflagellate cysts (i.e., dinocysts) are biologically and ecologically important, and many species’ cysts have protective cell walls that enable survival through harsh environmental conditions and facilitate their dispersal (Anderson & Wall, 1978; Dale, 1983). Dinocysts also serve as seeds for harmful algal blooms (HABs) (Anglès et al., 2012; Bravo & Figueroa, 2014). For example, dinocysts derived from *Alexandrium fundyense*, *Scrippsiella trochoidea* and *Cochlodinium polykrikoides* can cause severe HABs, resulting in a series of significantly deleterious effects on marine and coastal ecosystems, such as beach fouling, oxygen deficiency, and large-scale mortality of marine species (Bates & Trainer, 2006; Jeong et al., 2001; Shin et al., 2014; Park et al., 2013). In addition, it has been reported that many dinocysts such as those formed by *Alexandrium tamarense* contain higher toxin concentration than vegetative forms (Dale, 1983; Oshima, Bolch & Hallegraeff, 1992). The toxins produced accumulate along food webs and can cause significantly negative effects, even on human health (Anderson et al., 1996; Pan, Cembella & Quilliam, 1999; Kim et al., 2002; Kirkpatrick et al., 2004). Thus, the knowledge of density and distribution of dinocysts can permit the prediction of HABs (Bravo et al., 2006; Penna et al., 2010), facilitate deep understanding of population dynamics of dinoflagellates (Wyatt & Zingone, 2014; Anderson et al., 2014), and provide warning for toxin-associated diseases derived from sea food (Deeds et al., 2008; Tang & Gobler, 2012; Hattenrath-Lehmann et al., 2016). As the species identification represents the first and prerequisite step in many ecological studies, it is vital to accurately identify dinocysts to minimize their potential negative effects (Bravo et al., 2006; Kohli et al., 2014).

Dinocysts are traditionally identified based on available morphological characteristics, such as the shape of cyst body and its ornamentation, wall structure and color, and types of aperture or archeopyle (Matsuoka & Fukuyo, 2000). Morphological identification is successful for dinocysts with distinctive species-specific morphological features. However, many dinocysts cannot be identified at the species level mainly as available taxonomic keys do not always exist among related species, and/or available morphological features largely vary in the same species among different environments (Ellegaard, 2000; Matsuoka et al., 2009). For example, it is difficult to identify different morphotypes of cysts derived from the genera of *Gymnodinium* and *Alexandrium* (Wang, 2007; Bravo et al., 2006; Bolch, Negri & Hallegraeff, 1999). Alternatively, the corresponding vegetative forms are often used for species identification through germination of dinocysts (Gu et al., 2013; Ellegaard et al., 2003). Unfortunately, the germination of many dinocysts cannot be successfully achieved under laboratory conditions using current protocols (Tang & Gobler, 2012; Gu et al., 2013; Anderson et al., 2005). In addition, the accurate identification of species based on microscopic examination for both dinocysts and vegetative forms requires extensive taxonomic expertise. Owing to these inherent technical difficulties in morphological identification, molecular identification has become the first priority for dinocyst identification, although both methods should be cross-checked to ensure the identification accuracy.

Molecular methods, especially PCR techniques based on barcoding genes (i.e., barcoding method), have been developed and widely used for the identification of vegetative cells and dinocysts in both cultured and environmental samples (Bolch, 2001; Godhe et al., 2002; Godhe et al., 2007; Lin et al., 2006; Erdner et al., 2010; Penna et al., 2007; Penna et al., 2010). Molecular methods have many advantages when compared to traditionally morphological methods, and the most obvious one is that molecular methods have higher resolution on species-level identification (John et al., 2014; Du et al., 2011). However, the commonly used molecular identification largely relies on multiple dinocysts to get enough DNA to ensure successful PCR amplification (Penna et al., 2010; Bolch, 2001; Satta et al., 2013). The use of multiple dinocysts can easily result in wrong identification: although dinocysts have the same morphological feature based on microscopic examination, they may belong to different species as many dinocysts have similar even the same morphological features (Wang, 2007). Consequently, the use of single dinocysts in molecular identification is largely needed to improve the identification accuracy, and to establish barcoding databases for future molecular identification. Although Bolch (2001) developed a single cyst/cell-based method, such a method showed limited ability to identify single dinocysts due to faint bands or no bands on gels after PCR amplification, especially when used for heterotrophic species (Bolch, 2001). In addition, most species identified by molecular methods are plastid-containing dinoflagellate species, such as those from the genera of *Alexandrium* and *Gymnodinium* (Penna et al., 2010; Bolch, 2001; Godhe et al., 2002; Lin et al., 2006), and the identification of heterotrophic species by molecular methods has an extremely low success rate (Bolch, 2001).

The molecular identification of single dinocysts, especially heterotrophic species, is mainly hindered by technical issues associated with biological and/or genetic characteristics. Collectively, two major technical issues exist: (i) PCR inhibitors attached on the surface of dinocysts that are difficult to remove without any harm to dinocysts, and (ii) the lack of dionflagellate-specific PCR primers that can be used for single dinocyst identification. In order to minimize effects caused by PCR inhibitory substances contained in environmental samples such as those collected from marine sediments, possible solutions include the use of a commercial kit for DNA extraction and dilution of extracted DNA for PCR to reduce the quantity of inhibitors (Penna et al., 2010). However, these methods are not efficient enough to mitigate the negative effects caused by PCR inhibitors for single cyst identification, mainly owing to the extremely low quantity of template DNA for PCR amplification (Bolch, 2001). Molecular identification largely relies on the performance of barcoding genes and corresponding primers (Zhan et al., 2014), and heterotrophic dinoflagellate species may prey on other organisms such as diatoms (Bates & Trainer, 2006), which would be also amplified, and thus lead to failed identification of single dinocysts when using non-dinoflagellate-specific primers.

In this study, we aim to improve the success rate of single dinocyst identification. In order to remove contaminants attached on the cell wall of dinocysts, we optimized washing parameters for different types of dinocysts to efficiently remove contaminants but not to destroy the dinocyst structure. In addition, to improve the success ratio of molecular identification of single dinocysts, especially for heterotrophic dinoflagellate species, we designed new dinoflagellate-specific primers to increase the PCR efficiency.

## Materials and Methods

### Sediment sampling and processing

Sediment samples were collected using a benthic grab in one of the dinocyst hotspots (Jingdao, N21.52551° and E108.12738°) in the South China Sea in the summer of 2013. We sampled three times at three sampling sites (200 m apart), and for each sampling site, a total of 100 g sediment was collected and immediately transferred into black plastic bottles. All samples were stored at 4 °C during transportation and in the laboratory before analyses. For dinocyst checking and identification by both traditional morphological and molecular methods, all samples were treated according to the protocols by Matsuoka & Fukuyo (2000) and Wang (2007). Briefly, the sediments were well mixed before subsampling, and 5 g subsamples were weighed out and transferred into cleaned beakers. A total of 200 mL double distilled water ddH_2_O was added prior to ultrasonic wave treatment. The mixed water slurry was sieved successively through 125 µm and 20 µm meshes, and the sediment particles remaining on the 20 µm mesh were transferred into a beaker with 100 mL ddH_2_O and re-suspended and sonicated again using the recommended methods by Matsuoka & Fukuyo (2000) and Wang (2007). Subsequently, the treated suspension was sieved through a 20 µm mesh again, and the obtained particles were transferred into a pan and the upper light particles were pipetted out for single dinocyst isolation.

### Single dinocyst isolation, cleaning, and fragmentation

Each 0.5 mL particle suspension was pipetted onto a piece of clean slide and observed under Olympus CX-41 light microscope at a magnification of ×100–400. Once a single dinocyst, especially heterotrophic species, was found, small particles around the cyst were carefully separated away with sharp tweezers, and the dinocyst was transferred into a clean tube using a micropipette. In order to compare our optimized ultrasonic wave-based cleaning and traditionally used micropipette-based cleaning (Takano & Horiguchi, 2004), we divided the isolated dinocysts into two groups: the first group was treated using ultrasonic wave and the second group was treated using repeated pipeting up and down with micropipettes (Takano & Horiguchi, 2004). For the first group, 50 µL ddH_2_O were added into tubes containing isolated single dinocysts and then sonicated. As cell walls of different dinocysts have discrepant resistances to ambient pressure, the cleaning time was tested for different types of dinocysts to get the best cleaning results but not to break dinocysts. The lowest power of ultrasonic machine (50 Watt) was used mainly due to the little volume (50 µL) of ddH_2_O in tubes. The small volume (50 µL) of ddH_2_O was used in this study as larger amounts of water can easily lead to the loss of dinocysts during multiple processes.

Single dinocysts cleaned by the ultrasonic wave-based or traditionally used micropipette-based methods were pipetted onto a clean slide using a 10 µL micropipette, air-dried, and then covered with a clean glass slide and squeezed heavily to crush cells. The obtained contents were mixed with 10 µL ddH_2_O and immediately transferred into a 200 µL PCR tube. The obtained solution was used directly as the template DNA for PCR amplification.

### Primer design and PCR amplification

Although Lin et al. (2006) recommended a pair of dinoflagellate-specific primers for the amplification of environmental samples, the amplicon size is too large (1.6 kb). The large PCR amplicon is not easily amplified particularly when the amount of DNA is small, and easily makes subsequent sequencing and assembling troublesome and time-consuming, especially for bulk samples when using the state-of-the-art techniques such as high-throughput sequencing (Lin et al., 2009; Kohli et al., 2014). Consequently, we designed dinoflagellate-specific PCR primers based on the nuclear small subunit ribosomal DNA (SSU rDNA). SSU rDNA was used mainly because abundant data in public database such as GenBank can largely facilitate the species annotation after sequencing.

In order to design dinoflagellate-specific PCR primers, we retrieved sequences from NCBI GenBank for representative heterotrophic dinoflagellate species that can produce cysts. In addition, we retrieved sequences of representative species of three non-dinoflagellate groups (fungi, diatoms and green algae), which were frequently detected using eukaryote-universal primers to amplify single dinocysts, especially heterotrophic species. All downloaded sequences were aligned by ClustalW using default parameters implemented in MEGA version 6.0 (Tamura et al., 2013) to select target fragments for PCR primer design (Appendix S1). The fragments were chosen for primers design mainly based on the following standards: the primer annealing sites are conserved among different dinoflagellate species but variable between dinoflagellate and non-dinoflagellate species, while the fragments between primer annealing sites are hypervariable among dinoflagellate species to ensure species delimitation with a high resolution power. Dinoflagellate-specific PCR primers were designed using the Premier primer 5.0 (Lalitha, 2000) and BioEdit 7.0 (Hall, 1999). To test the performance of the newly designed primers, we tested the PCR amplification efficiency and specificity using single dinocysts cleaned by ultrasonic waves based on our optimized parameters.

PCRs were performed on a Mastercycler nexus (Eppendorf, Hamburg, Germany) in a 25 µL PCR mix containing 1 × *Ex Taq* Buffer (20 mM Mg^2+^ plus, Takara, Dalian, China), 5.0 mM of each dNTP, 15 pmol forward and reverse primers, 0.75 U of *TaKaRa Ex Taq* (Takara, Dalian, China), and the following cycle conditions: 95 °C for 5 min; then 35 cycles at 95 °C for 30 s, 55–59 °C for 45 s determined based on different primers (Table 1) and 72 °C for 30 s; and a final extension at 72 °C for 7 min.

**Table 1:** Newly designed primers based on the nuclear small subunit ribosomal DNA (SSU rDNA) in this study.

Primer name	Sequences (5′→3′)	Amplicon length (bp)	*T*_*a*_ (°C)
18S286F	GTCCGCCCTCTGGGTG	286	55
18S286R	TCGCAGTAGTSYGTCTTTAAC		
18S468F	GAAATAACAATACARGGCATCCAT	468	59
18S468R	TTCGCAGTAGTCCGTCTTTAAC		
18S489F	TGGCCGTTCTTAGTTGGTG	489	57
18S489R	TGTTACGACTTCTCCTTCCTCT		
18S493F	CATGGCYGTTCTTAGTTGGTG	493	57
18S493R	TGTTACGACTTCTCCKTCCTCT		
18S634F	GGGTAACGGAGAATTAGGGTTT	634	59
18S634R	TCCCCTAACTTTCGTTCTTGATC		

**Figure 1:**
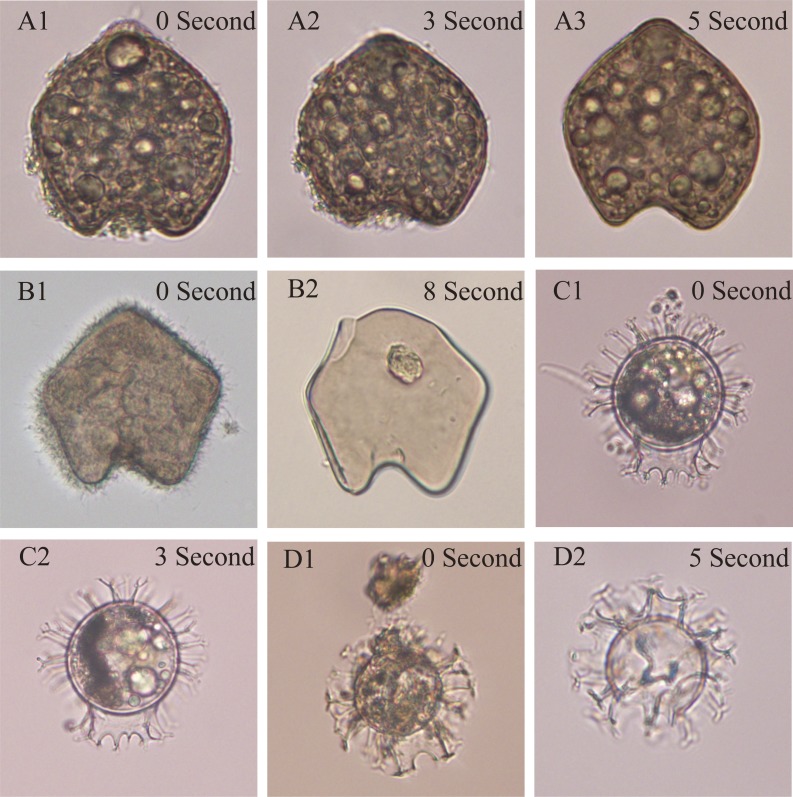
The test of optimized ultrasonic cleaning time on two typical species belonging to two major groups (*Protoperidinium* and *Gouyaulax*). A1-3 and B1-2, and C1-2 and D1-2 show the same species: * Protoperidinium oblongum* and *Gouyaulax spinifera*, respectively, but were treated with different ultrasonic cleaning times.

### Species identification

The success of PCR was checked on 2% agarose gels. The successful amplicons were sequenced using a Big Dye Terminator cycle sequencing kit on an ABI PRISM 3730 Genetic Analyser (Applied Biosystems, Foster City, CA, USA). The obtained sequences were identified using BLASTN against the NCBI GenBank database.

## Results

### Cleaning parameters

Our results showed that the cleaning time of 3 s was appropriate for the cysts formed by the genera of *Gonyaulax*, *Polykrikos*, *Lingulodinium*, *Pyrophacus*, and 5 s for the *Protoperidinium* group (Fig. 1). When the cleaning time was less, the impurities adhering to dinocysts were not cleaned completely (Fig. 1A2); however, broken dinocysts were frequently observed when the cleaning time was longer (Figs. 1B2, 1D2).

**Figure 2:**
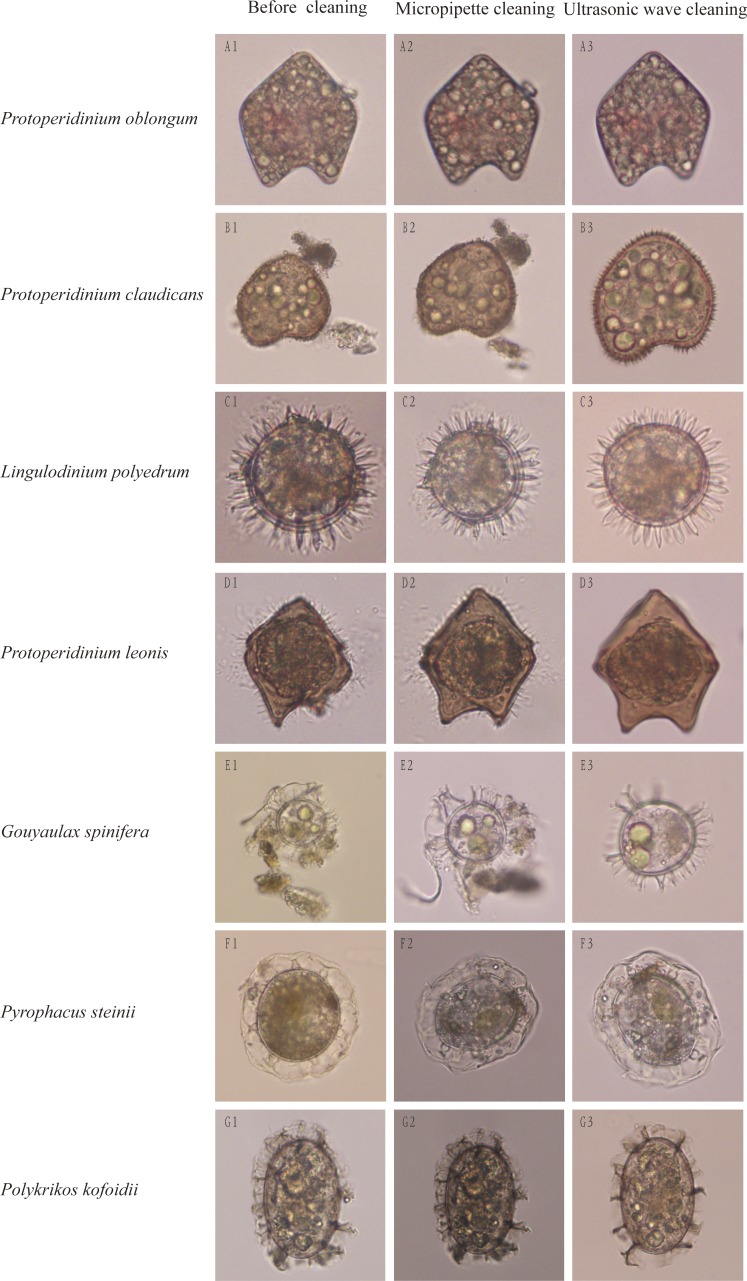
Cleaning effects on dinocysts by traditional micropipettes cleaning or our optimized ultrasonic waves-based cleaning. The left rows are dinocyst species names and the top of columns are cleaning methods.

Impurities surrounding dinocysts of different species were largely removed using the method of optimized ultrasonic wave-based cleaning at respective optimized cleaning parameters. After cleaned by the ultrasonic wave, surface structures of single cysts were easily distinguishable and can be used for morphological identification (Fig. 2). For example, before cleaning, the dinocyst (Fig. 2B1) could be identified as *Protoperidinium oblongum* or *P. claudicans* based on the traditional morphological identification, but this cyst can be accurately identified as *P. claudicans* after cleaning as many short processes appeared on the cyst (Fig. 2B3). Similarly, the cyst surface was not smooth before cleaning as many fungus-like impurities attached on its surface (Fig. 2D1). The fungus-like impurities were easily identified as short processes, leading to an incorrect identification, whereas these fungus-like impurities were thoroughly removed after cleaning and the correct identification became reliable based on morphological characteristics (Fig. 2D3).

### Primer design and test

We designed five pairs of dinoflagellate-specific primers (Table 1). When the primer pairs 18S468F-18S468R, 18S489F-18S489R and 18S493F-18S493R were used to amplify single dinocysts of the genera of *Gonyaulax*, *Polykrikos* or *Protoperidinium*, failed PCR amplifications were often detected (Table 2). However, both 18S286F-18S286R and 18S634F-18S634R could successfully amplify all dinoflagellate species that we isolated and tested, and we detected clear and sharp bands on gels after PCR amplification (Table 2). Finally, the primer pair 18S634F-18S634R was selected for the molecular identification of single dinocysts.

### Molecular identification

A total of 348 dinocysts were observed and identified by traditional morphological identification before cleaning. Dinocysts with distinctive morphological features and/or identified as different species were used for further analysis. Finally, 42 dinocysts including 28 heterotrophic dinocysts and 14 plastid-containing dinocysts (Table 3) were selected for the comparison of molecular identification based on traditional micropipette cleaning and our optimized ultrasonic wave cleaning. One group, including 12 dinocysts (belonging to the genera of *Protoperidinioid*, *Gymnodinioid*, *Tuberculodinioid* and *Gonyaulacoid*), was subjected for cleaning using micropipettes, the other group, including 30 dinocysts (belonging to the genera of *Protoperidinioid*, *Gymnodinioid*, *Tuberculodinioid* and *Gonyaulacoid*), was cleaned using the optimized ultrasonic wave method (Table 3).

**Table 2:** Preliminary tests for all primers designed in this study based on single dinocysts.

Primers	Dinoflagellate species					
	*Gouyaulax spinifera*	*Lingulodinium polyedrum*	*Pyrophacus steinii*	*Polykrikos kofoidii*	*Protoperidinium leonis*	*Protoperidinium oblongum*
18S286F–18S286R	Clear band	Clear band	Clear band	Clear band	Clear band	Clear band
18S468F-18S468R	No band	−	−	−	No band	No band
18S489F-18S489R	No band	No band	Faint band	No band	−	Clear band
18S493F-18S493R	No band	−	No band	No band	Faint band	No band
18S634F-18S634R	Clear band	Clear band	Clear band	Clear band	Clear band	Clear band

**Notes.**

“−” indicates that primers were not tested for corresponding dinocysts due to the limited amount of isolated single dinocysts.

**Table 3:** Comparisons of effects on the molecular identification of single dioncysts by different cleaning methods.

Sample number	Cleaning methods	Morphology identification	Trophic type	Molecular identification	Success rate
1	Micropipette	*Gouyaulax* sp.	Plastid-containing	Failure	16.70%
2		*Gouyaulax spinifera*	Plastid-containing	Failure	
3		*Lingulodinium polyedrum*	Plastid-containing	Failure	
4		*Pyrophacus steinii*	Plastid-containing	*Pyrophacus steinii*	
5		*Polykrikos kofoidii*	Heterotrophic	Failure	
6		*Polykrikos kofoidii*	Heterotrophic	*Polykrikos kofoidii*	
7		*Polykrikos kofoidii*	Heterotrophic	Failure	
8		*Protoperidinium leonis*	Heterotrophic	Failure	
9		*Protoperidinium leonis*	Heterotrophic	Failure	
10		*Protoperidinium oblongum*	Heterotrophic	Failure	
11		*Protoperidinium oblongum*	Heterotrophic	Failure	
12		*Protoperidinium oblongum*	Heterotrophic	Failure	
13	Optimized ultrasonic wave cleaning	*Gouyaulax spinifera*	Plastid-containing	*Gouyaulax spinifera*	86.70%
14		*Gouyaulax* sp.	Plastid-containing	*Gonyaulax cochlea*	
15		*Gouyaulax spinifera*	Plastid-containing	Failure	
16		*Gouyaulax spinifera*	Plastid-containing	Failure	
17		*Gouyaulax spinifera*	Plastid-containing	*Gouyaulax spinifera*	
18		*Gouyaulax spinifera*	Plastid-containing	*Gouyaulax spinifera*	
19		*Gouyaulax spinifera*	Plastid-containing	*Gouyaulax spinifera*	
20		*Lingulodinium polyedrum*	Plastid-containing	*Lingulodinium polyedrum*	
21		*Lingulodinium polyedrum*	Plastid-containing	*Lingulodinium polyedrum*	
22		*Pyrophacus steinii*	Plastid-containing	*Pyrophacus steinii*	
23		*Polykrikos kofoidii*	Heterotrophic	*Polykrikos kofoidii*	
24		*Polykrikos kofoidii*	Heterotrophic	*Polykrikos kofoidii*	
25		*Polykrikos kofoidii*	Heterotrophic	*Polykrikos kofoidii*	
26		*Protoperidinium claudicans*	Heterotrophic	*Protoperidinium claudicans*	
27		*Protoperidinium leonis*	Heterotrophic	*Protoperidinium leonis*	
28		*Protoperidinium leonis*	Heterotrophic	*Protoperidinium leonis*	
29		*Protoperidinium leonis*	Heterotrophic	*Protoperidinium leonis*	
30		*Protoperidinium leonis*	Heterotrophic	*Protoperidinium leonis*	
31		*Protoperidinium leonis*	Heterotrophic	*Protoperidinium leonis*	
32		*Protoperidinium leonis*	Heterotrophic	Failure	
33		*Protoperidinium leonis*	Heterotrophic	*Protoperidinium leonis*	
34		*Protoperidinium leonis*	Heterotrophic	*Protoperidinium leonis*	
35		*Protoperidinium oblongum*	Heterotrophic	*Protoperidinium oblongum*	
36		*Protoperidinium oblongum*	Heterotrophic	*Protoperidinium oblongum*	
37		*Protoperidinium oblongum*	Heterotrophic	*Protoperidinium oblongum*	
38		*Protoperidinium oblongum*	Heterotrophic	Failure	
39		*Protoperidinium oblongum*	Heterotrophic	*Protoperidinium oblongum*	
40		*Protoperidinium oblongum*	Heterotrophic	*Protoperidinium oblongum*	
41		*Protoperidinium oblongum*	Heterotrophic	*Protoperidinium oblongum*	
42		*Protoperidinium oblongum*	Heterotrophic	*Protoperidinium oblongum*	

When the cleaning effects were compared between the two methods for all dinocyst species, the success rate was 86.7% for our optimized ultrasonic wave-based method, which was much higher than that (16.7%) of the micropipette method (Table 3). After being cleaned by ultrasonic wave, 26 of 30 dinocysts were successfully identified using the dinoflagellate-specific primers (18S634F-18S634R; Table 3). However, only 2 of 12 dinocysts cleaned by micropipettes belonging *Polykrikos kofoidii* and *Pyrophacus steinii* were identified successfully after cleaned by micropipettes. Out of 20 heterotrophic dinocysts, 18 dinocysts (90%) were successfully identified based on the method improved in this study (Table 3). In addition, 8 of 10 (80%) plastid-containing dinocysts were also successfully identified (Table 3).

For each dinocyst species, the cleaning effects of two cleaning methods were also compared (Table 4). We failed to identify all *Gouyaulax spinifera*, *Gouyaulax* sp., *Lingulodinium polyedrum, Protoperidinium leonis and Protoperidinium oblongum* cysts cleaned by micropipettes. However, the success rates of those dinocysts cleaned by our optimized ultrasonic cleaning method were 66.7%, 100%, 100%, 87.5% and 87.5%, respectively, much higher than those based on micropipette cleaning. For *Polykrikos kofoidii*, the success rate based on micropipette cleaning was 33.3%, while the optimized ultrasonic cleaning method improved the success rate to 100%.

**Table 4:** The comparison of identification success rates of each dinocyst species cleaned by different methods.

Species	Success rates	
	Micropipette (%)	Ultrasonic wave (%)
*Gouyaulax spinifera*	0	66.7
*Gouyaulax* sp.	0	100
*Lingulodinium polyedrum*	0	100
*Pyrophacus steinii*	100	100
*Polykrikos kofoidii*	33.3	100
*Protoperidinium leonis*	0	87.5
*Protoperidinium oblongum*	0	87.5

## Discussion

Dinocysts isolated from environmental samples such as sediment often contain many contaminants, which largely influence both morphological and molecular identifications (Wilson, 1997; Miller, 2001). When compared to both non-cleaned and micropipette-cleaned dinocysts in this study, the cleaned dinocysts by our optimized ultrasonic wave-based method clearly showed that contaminants attached on the surface of dinocysts largely affected the observation of micro-structures, many of which are considered as taxonomic keys for morphological identification. Our results showed that the cleaning time differed among different taxonomic groups: when the power of ultrasonic wave was 50 Watt and the volume was 50 µL, three seconds were appropriate for the species in the genera of *Gonyaulacoid*, *Gymnodinioid* and *Tuberculodinioid*, and five seconds for the *Protoperidinioid* group. When the ultrasonic wave-based method for single dinocysts was used, contaminants attached on dinocysts were largely removed, and the cleaned dinocysts were easily identified by both morphological and molecular identifications.

Our molecular identification results suggest that the contaminants attached on dinocysts could be PCR inhibitors in the molecular identification. Those contaminants can lead to failure in PCR by preventing the amplification of nucleotide fragments interacting with nucleotide molecules or inhibiting *Taq* polymerase activities (Bessetti, 2007; Wilson, 1997; Peist et al., 2001). The major source of PCR inhibitors can be divided into two groups: attached debris and introduced chemicals (Wilson, 1997). PCR inhibitors from the former are complex and numerous, such as humic acid, polysaccharides and various tiny organisms, while PCR inhibitors from the latter mainly include dodium deoxycholate, sarkoysyl, sodium dodecyl sulfate, ethanol, isopropanol and phenol derived from DNA extraction procedures. PCR inhibitors from chemicals introduced during DNA extraction were not applied here, as we did not use the chemical-based DNA extraction method. Chemical free methods are recommended in the molecular identification of single dinocysts, especially those derived from sediments containing various PCR inhibitors (Miller, 2001). Thus, the major inhibitors in this study came from attached debris on the surface of dinocysts.

In order to improve the success ratio of molecular identification, we newly designed five pairs of primers, two of which (18S634F-18S634R and 18S286F-18S286R) showed a high degree of amplification efficiency (86.7% success ratio). Indeed, the high success ratio also suggests a high level of specificity of these primers, as the failure of amplification of target dinocysts can largely decrease the success ratio. The high level of specificity is reserved by the strategy used in the process of primer design (both dinoflagellates and non-dinoflagellates were considered). Based on our data, all successfully amplified dinocysts were identified as dinoflagellates, and the 13.3% failed cases were due to PCR failure (Table 3). Moreover, the amplicon length (286bp) of the primer pair 18S286F-18S286R makes it a good candidate for high throughput sequencing-based large-scale analyses of environmental samples.

Heterotrophic dinoflagellates, which can prey on other organisms, play key roles in food webs (Jeong et al., 2010). More importantly, some heterotrophic dinoflagellates, such as *Polykrikos kofoidii*, have the potential to be used for the control of HABs (Bates & Trainer, 2006; Jeong et al., 2001; Jeong et al., 2003). However, heterotrophic dinocysts are not easy to be identified due to two major reasons. Firstly, many species, especially closely related ones, have largely similar morphological features. For example, the species belonging to *Brigantedinium* have common morphological features as “brown round shape” and cannot be identified based on morphological features (Matsuoka et al., 2006; Li et al., 2015). Secondly, it is extremely difficult to germinate heterotrophic dinocysts due to their heterotrophic nature. Although great efforts have been made for the molecular identification of single heterotrophic dinocysts, successful cases are scarce when compared with large efforts made on identification (Bolch, 2001). The results in this study clearly showed that our improved method could identify heterotrophic dinocysts with a high success rate (90%). The improved method here would provide a new molecular approach for the identification of heterotrophic dinocysts.

In conclusion, the methods that we improved here are technically simple but can efficiently identify both single heterotrophic and plastid-containing dinocysts. Collectively, two technical issues that we addressed here were largely responsible for the high success rate: the dinoflagellte-specific primers designed in this study can efficiently amplify the target DNA fragments, while possible PCR inhibitors attached on dinocysts were largely removed using our optimized ultrasonic wave-based cleaning method. Both improved technical issues here ensure the successful amplification of single dinocysts under the condition of a small quantity of the total DNA.

##  Supplemental Information

10.7717/peerj.3224/supp-1 Appendix S1 Sequences used for primer design

## Additional Information and Declarations

### Data Availability

The following information was supplied regarding data availability: The raw data has been supplied as a Supplementary File.
